# Assessing artificial intelligence’s impact on e-customer loyalty in the Saudi Arabian market

**DOI:** 10.3389/frai.2025.1541678

**Published:** 2025-04-30

**Authors:** Hasan Beyari

**Affiliations:** Department of Administrative and Financial Sciences, Applied College, Umm Al-Qura University, Makkah, Saudi Arabia

**Keywords:** artificial intelligence, e-customer loyalty, social media exposure, product recommendation, brand preference, purchase intention

## Abstract

This study investigated the effect of artificial intelligence on e-customer loyalty in the Saudi Arabian e-commerce market. It evaluates five important variables: social media exposure, product recommendation, brand preference, purchase intention, and e-customer loyalty. The study espoused primary research methodologies by employing a questionnaire and surveying East, West, and Central Saudi Arabia. The sample size was 157 respondents, a blend of males, females, and persons of all ages. We developed a structural equation model based on six hypotheses. Ultimately, the study provided evidence that led to the confirmation of the hypotheses. We obtained credible scores in assessing the measurement model where we considered indicator reliability (0.920), internal consistency—Cronbach’s alpha (0.902), and convergent reliability – measured by Average Variance Extracted (0.765). The model fit indices indicated the model’s chi-square score was 514.355 and a CMIN/DF of 3.117. The study found that AI, particularly social media exposure and product recommendations, strongly influences Saudi e-customer loyalty. The positive association between social media exposure, purchase intention, and brand preference reveals how focused material affects customer behavior. We conclude that the model is statistically significant and that all hypotheses are supported. The implication is that artificial intelligence is a valid strategy for attaining customer loyalty on e-commerce platforms.

## 1 Introduction

The Saudi e-commerce market is among the most developed in the MENA region. Recent statistics by Tulcanaza-Prieto et al. (2023) suggest that the market grew to $11.83 billion. The same source indicates that this value will further burgeon to $23.80 billion by the end of 2028. As a result, the industry plays a critical role in developing the local and regional economy. At the same time, some analysts argue that the sector received an unpopular boost during the COVID-19 pandemic because of restrictions on people’s movement. Recent trends indicate that the growth is still sustainable in the future (Goti et al., 2023). Several new e-commerce businesses have emerged, and the incumbent ones have expanded further to cater to previously un-covered populations. New features like home and office delivery are becoming more prominent as the industry grows.

One central phenomenon businesses have in common is their preference to adopt artificial intelligence, especially in social media data and product recommendation engines. Between 2020 and 2023, artificial intelligence (AI) in the sector has supported about 60% of the sales recorded in Saudi Arabia. As noted by Helmi et al. (2024), the industry favors businesses adopting these technologies. AI is a technological field that constructs models to imitate and forecast human behavior. About five of every six e-commerce rely on artificial intelligence to enhance customer experience or boost sales. Integration of AI tools has significantly contributed to a surge in online sales, particularly in apparel, electronics, and appliances. Fortunately for the market, Saudi’s technological infrastructure highly supports AI development by businesses. According to Jenneboer et al. (2022), the e-commerce sector has created tech jobs for more than 30,000 people who build, test, run, and deploy machine and deep learning models in the country. The trend reflects the world’s steady adoption of artificial intelligence, given the growing popularity of new-age AI models such as GPT and BERT.

The main reason for the consistently rising uptake of AI technologies among e-commerce businesses in Saudi Arabia is its potential to induce market growth. The shift in consumer behavior toward online purchases of convenience and shopping products has also justified using artificial intelligence to improve user experience and encourage more sales (Marjerison et al., 2022). The conventional in-store shopping experience, which is slowly becoming unpopular, has long been successful partly because of human intervention to assist those stuck. The digital migration meant no direct human touch between the buyer and the business. As Bhagat et al. (2023) asserts, artificial intelligence fills this gap by acting as a human assistant. Many e-commerce businesses have AI assistants in chat applications integrated into the shopping platforms (Yılmaz Benk et al., 2022). Given the complexities typical around online shopping, having these assistants ready whenever customers need help with delayed deliveries, finding rare products, and general site navigation is prudent.

This research analyzes AI integration in both website-based and mobile application e-commerce platforms because these platforms now extensively use AI technologies. The frequent contact between mobile e-commerce users and AI-generated content again becomes more pronounced when users interact through social media (Huang et al., 2023). Every consumer uses smartphones to access social media; thus mobile platforms organize the core channel for time-sensitive product recommendations alongside targeted influencer marketing alongside customized push notifications. Most technologies that run on websites depend on AI technologies, just like mobile applications; however, they frequently lack the emotional connection and instant delivery that mobile applications provide (Al-Mushayt et al., 2022). The research design includes both contexts to represent how contemporary consumers interact with digital technologies yet recognizes that AI functions become more intense and operate differently between website and mobile platforms.

Leading e-commerce platforms, including Noon, Amazon.sa, and Jarir Bookstore Online, use artificial intelligence in Saudi Arabia with sophisticated regional consumer-based technology solutions (Al-Mushayt et al., 2022). The recommendation engines at Noon use artificial intelligence to process website patterns and purchase history data while tracking Saudi Arabian shopping trends to deliver exact recommendations (Helmi et al., 2024). The platform connects product recommendation features to its mobile interface and desktop version, producing instant promotion recommendations for customers to increase engagement rates and drive unchecked purchases. The AI-based pricing models at Noon automatically calculate product values based on user actions, periods, and market activity to create perceived customer value (Daou and Stephan El Khoury, 2025).

The Saudi Arabian branch of Amazon works as a part of Amazon’s worldwide system that applies proprietary machine learning programming to boost customer contentment. The recommendation system “*Customers who bought this also bought…*” operates as one of its core features (Al-Mushayt et al., 2022). The application uses collaborative filtering models to examine big customer data sets. The Middle Eastern market receives personalized bundle offers and seasonal promotions from Amazon.sa specifically designed to satisfy Saudi customer needs during major shopping periods, including Ramadan and White Friday (Daou and Stephan El Khoury, 2025). The features powered by AI technology act as essential tools that stimulate frequent buys and enhance customer commitment to the platform. Jarir Bookstore presented a mix of physical stores and digital features containing AI technology for its service chatbots, search results optimization, and behavioral assessment to produce enhanced promotional approaches (Helmi et al., 2024). These examples show that AI plays a strategic role in Saudi e-commerce systems since it creates deeper customer connections through customized services, instant support, and ease of use.

E-commerce businesses are obsessed with customer loyalty because of its potential to rump up sales through repeat and referral purchases. Saudi e-commerce firms are not an exception to this rule, striving to ensure a loyal customer base (Rana et al., 2022). These entities have applied several methods and techniques, such as points-based, value-based, paid, and tiered loyalty programs. Their benefits have been better customer engagement, sales and revenue growth, quantifiable impact on brand affinity, and a significant reduction in customer churn rate (Ahmad and Akbar, 2023). These methods have not only been difficult to implement but have also been costly. The advent of artificial intelligence has simplified the process and endeavored to enhance customer engagement more conveniently from the perspectives of businesses’ customers. On the one hand, customers are more likely to leave positive feedback as they check out because of the enhanced experience (Fedushko and Ustyianovych, 2022). On the other hand, businesses do not have to spend much on customer care staff, as AI technology is smart enough to understand customer preferences.

This investigation aims to measure the effect of artificial intelligence on e-customer loyalty among e-commerce platforms in Saudi Arabia. The research approach is reasonably nuanced, as it does not simply seek to investigate the direct relationship between AI and e-customer loyalty. Instead, it appreciates these variables’ complicated relationship with critical factors, namely brand customer purchase intention and preference. Few articles touch on all these factors and build a model to explain consumer behavior from this perspective. Hence, the research uniquely portrays the relationships between and among AI, purchase intention, brand preference, and e-customer loyalty. In achieving this objective, the paper begins with a substantive literature review, reviews the theoretical background, constructs the methodology, presents the results, and discusses them in light of previous studies.

## 2 Literature review

Artificial intelligence relies heavily on social media exposure to interact with potential and current e-commerce customers. Without this exposure, Bilal et al. (2024) argues that the value of artificial intelligence would diminish. The relationship between social media exposure and purchase intention is reasonably dynamic. Bhagat et al. (2023) finds a 40% increase in customers’ purchase intention with a month-long exposure to social media. Firms that engage heavily in social media activity tend to front such studies as their justification. Nevertheless, Onofrei et al. (2022) finds no significant difference in customers’ purchase intentions when exposed or not exposed to social media (Anantasiska et al., 2022). The study argues that social media does not necessarily contribute to purchase intention because different users find it engaging at different levels. This view is consistent with Foroughi et al. (2024), who finds that user engagement in social media is the aspect that correlates with customer purchase intention. The study finds that when users find social media content engaging enough, they tend to experience a 25% increase in their purchase intention of the products associated with the social media content with which they have interacted (Han and Balabanis, 2024).

Another aspect of artificial intelligence that has been the focus of scholarly investigation regarding its effect on purchase intention is product recommendation Bhagat et al. (2023) finds that product recommendation engines are responsible for about 70% of impulse buying instances in online shopping. Such recommendations work best when a customer searches, favorites, likes, or purchases a specific product. In this case, the engine recommends similar or complementary products. Deeper models can even suggest other products in which similar customers have shown interest. The conversion rate of products recommended using AI is 30% higher than when not using the technology (Jin and Zhang, 2025). Nevertheless, Revilla et al. (2023) opines that companies over-relying on AI recommenders risk overwhelming customers with viable options, leading to a phenomenon known as over-choice. It is a situation in which a customer fails to go through with a specific purchase because of too many available options.

Social media exposure can also be instrumental in influencing customers’ brand preferences (Maskuroh et al., 2022) considers content targeting to be social media’s most formidable arsenal in influencing consumer tastes and preferences. A site like Facebook allows advertisers to target users of a specific demographic quality, including their age, profession, or interests. In one study by Bhattacharya (2023), content targeting influenced customer brand preference by 45% and click-through rate by about 20%. These statistics explain why many e-commerce companies push their marketing content on social media (Liao et al., 2021) cautions that AI-driven content can work against a brand if there is a misalignment between a user’s preferences and the model’s suggestions. In other words, if they get it wrong, the customer may even go off social media because it shows them options they have no interest in. Despite this risk, several studies agree that consistent brand messaging on social media platforms solidifies customer preference positions. For this reason, this technique remains popular among small and major e-commerce businesses across the globe.

Some studies have suggested a significant relationship between product recommendation and brand influence or preference. Kim et al. (2021) found that effective recommendation algorithms can improve brand preference for 60% of users. Similarly, Agustian et al. (2023) found that a significant increase in brand preference is correlated with accurate product recommendations, especially among tech-savvy users. This improvement highlights the potential of artificial intelligence to influence consumer perceptions positively. However, Khoirunnisa and Albari (2023) warns against the negative consequences of assertive AI product placements. The study finds that 15% of users may feel overwhelmed, potentially diminishing brand preference. These findings show the dynamics of customizing AI strategies to the intended audience’s technological expertise. The approach ensures that the AI’s assertiveness is suitable and effective. While there are risks to relying on product recommendations in influencing customer brand preference, e-commerce businesses seem devoted to trusting the algorithms (Cheng and Jiang, 2022). Consequently, many such businesses have implemented measures to curb AI’s assertiveness.

Research on how purchase intention impacts e-customer loyalty has also been rife, with many studies suggesting a positive relationship. Ahmad and Akbar (2023) write on this relationship and report that strong purchase intention translates to a higher likelihood of repeat purchases. The study further asserts that e-commerce platforms capitalize on their customers’ purchase intentions to ensure that customers remain loyal to the companies and their products. Machi et al. (2022) argues that e-customer loyalty is only moderately affected (20% increase) by purchase intention when not supported by customer satisfaction. The article acknowledges that customer purchase intention can be difficult to measure. However, if correctly measured, it can result in loyalty. On the contrary, Zhang and Ahmad (2021) argues that purchase intention due to seasonal sales does not significantly affect long-term e-customer loyalty. This review shows the multi-faceted relationship between purchase intention and e-customer loyalty.

Like purchase intention, brand preference has also received considerable attention from scholars regarding its effect on e-customer loyalty. Pu et al. (2025) report a direct correlation where strong brand preference boosts customer loyalty. The study is unclear about the measured effect but argues that there is a significant difference between the loyalty of customers with strong brand preferences and those with weaker versions of this preference. According to Valmohammadi et al. (2025), loyalty programs are more effective (60% increase in loyalty) when aligned with existing brand preferences. The overall suggestion is that when adequately considered by management, brand preference can be a strategic tool in advancing a business’s endeavors to influence customer loyalty. This view is, however, in contrast to what Zaato et al. (2023) finds. The source acknowledges the importance of brand preference but would not go as far as linking it to customer loyalty. Specifically, the article argues that customer loyalty is not significantly influenced by brand preference in highly competitive markets due to price sensitivity. Hence, there seems to be a lack of consensus on the relationship between the two variables, but the evidence leans toward a positive causal relationship.

Previous research correctly emphasized COVID-19 pandemic-induced e-commerce increases, yet modern academic work shows consumers have shifted their loyalties because of coronavirus-related changes. Campisi et al. (2023) expanded their expectations across personalization, convenience, and post-purchase engagement. AI has evolved by improving recommendation tools and sentiment-tracking chatbots, focusing on developing lasting customer relationships instead of basic transaction efficiency. The research by Agarwal et al. (2024) establishes that improved emotional engagement metrics for e-customers can now be accurately measured due to AI applications in customer journey mapping technologies. Companies now require advanced dynamic AI models to handle complex post-COVID customer segmentation since economic behavior patterns, remote work preferences, and trust levels have become unsteady (Cheng et al., 2023). The transformation of AI serves as a clear indication that customer loyalty development through AI has advanced beyond easy convenience mechanisms into becoming the central tool producing trust and emotional connections in digital platforms.

Recent research shows customers have developed new privacy-related anxieties that surpass the traditional concerns about artificial intelligence functionalities (Noble and Mende, 2023). AI remains at the core of improving personalization and convenience on platforms. Yet, some consumers now avoid AI-driven systems because they sense increased data risks, want to see algorithmic clarity, and want to overcome digital stress (Aldboush and Ferdous, 2023). A visible change in user behavior demonstrates an avoidance of automated interactions that they consider too intrusive or whose data practices remain clouded. Manufactured by ethical AI discussions and worldwide discussions on surveillance capitalism and algorithmic bias, consumers have developed a critical approach that combines self-preservation and greater protection (Aldboush and Ferdous, 2023). Organizations continue to develop AI strategies that simultaneously serve efficiency purposes while providing visible operations, maintaining ethical principles, and providing value-based solutions because digital trust is becoming more vulnerable (Bhattacharya et al., 2023). Businesses face an obligation to explain recommendation system processes to their customers and provide them with data access privileges while maintaining AI outputs that match personal values. Today’s customer loyalty depends on gaining extended trust in a marketplace that carefully watches data usage and society’s social trends.

Following the pandemic, consumer behavior has developed profound psychological and behavioral changes along with technological advancements. The pandemic accelerated consumer value-oriented behavior because people now focus on essential products, safe transactions, and cost transparency instead of product variety (Campisi et al., 2023). The rapid pandemic-driven digitization revealed numerous privacy issues to end-users, which sparked increased attention to AI system data handling and deployment methods (Cheng et al., 2023). Rising digital awareness creates digital resilience that makes people suspend trust automatically, even for highly convenient e-commerce platforms (Al-Ayed, 2022). The post-COVID world defines customer loyalty through platforms demonstrating ethical data management practices and their ability to exhibit digital ethics (Helmi et al., 2024). User expectations for digital agencies demand that AI systems achieve personalized services without compromising privacy or requiring users to relinquish control over their data (Qin et al., 2022).

## 3 Theoretical background

### 3.1 Technology acceptance model (TAM)

The study is partly anchored in the technology acceptance model. The perceived usefulness of the e-commerce platform has been enhanced due to AI’s impact on the online purchasing experience (Saleem et al., 2022). This factor plays into the equation of balancing a technology’s complexity against its usefulness. In this case, useful technology is more useful than complex technology. Artificial intelligence makes e-commerce systems simpler and more useful, making them a key addition to the technology ecosystem. Another key aspect of TAM is acceptance by users. The acceptance and utilization of e-commerce services may be bolstered by the perceived simplicity of use associated with AI-driven recommendation systems. This feature simplifies the process and makes it more engaging and sometimes fun. Ganoune et al. (2023) finds that systems inducing a good level of engagement with its users is more likely acceptable to them. Their positive experiences with artificial intelligence may positively influence the inclination of Saudi consumers toward e-commerce platforms.

The reinforcement of users’ intention to utilize e-commerce may result from their acknowledgment of AI’s advantages. These advantages are mostly in product recommendations and social media exposure, as hypothesized in this study. In the Saudi market, actual usage rates of AI-integrated e-commerce platforms may be predicted by a combination of high perceived usefulness and simplicity of use. Saleem et al. (2022) argues that external variables, including consumer confidence in artificial intelligence and privacy considerations, may impact the constructs of TAM. Moreover, the state of the digital infrastructure in Saudi Arabia may also influence TAM constructs. User attitudes and intentions toward e-commerce platforms may be influenced by subjective norms such as social influence and peer recommendations (Ganoune et al., 2023). AI potentially amplifies these variables.

The potential influence of perceived ease of use on actual usage may be moderated by the degree of users’ familiarity with AI and the voluntary nature of their interactions with it. An additional factor that may impact AI’s perceived usefulness and simplicity of consumer feedback regarding their experiences with the technology is the alterations in technological proficiency and age (Habib and Hamadneh, 2021). Diverse demographic groups may experience contrasting effects of AI on TAM constructs, which could have implications for the general acceptance of technology.

### 3.2 Theory of reasoned action (TRA)

The theory of reasoned action is another theoretical pillar anchoring this study. It asserts that attitudes and behaviors impact an individual’s actions (Saleem et al., 2022). Regarding behavioristic beliefs, the Saudi consumer’s perception of online purchases may be positively impacted by the belief that AI improves purchasing efficiency. This belief is further reinforced if evidence from past interactions and other people’s experiences attests to it. An important consideration in e-commerce that potentially influences consumer behavior in the e-commerce sector is the recommendation engine (Warganegara and Babolian Hendijani, 2022). Consumers may develop favorable attitudes toward the behavior if they perceive value in the recommendations generated by artificial intelligence and the exposure they receive on social media. Societal norms and the perception of AI in Saudi culture may impact consumers’ attitudes and subjective norms. Social circles’ approval of artificial intelligence in e-commerce may generate normative pressure to participate in online purchasing (Lau et al., 2023). Positive attitudes and perceived social pressure regarding using AI-enhanced e-commerce services may increase purchase intentions. The anticipation of utilizing AI-driven electronic commerce platforms will likely result in tangible buying actions.

### 3.3 Conceptual model

**Figure 1:**
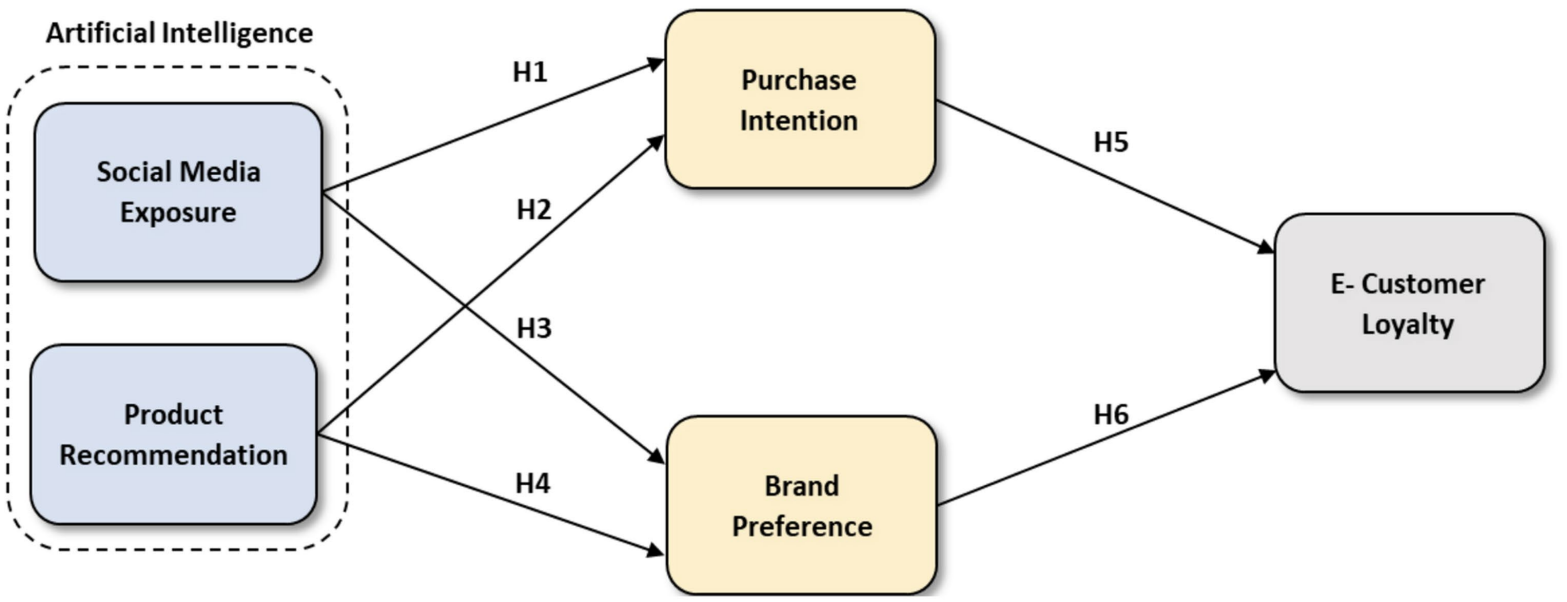
Study’s conceptual model.

The diagram below shows the interactions between and among study variables. The artificial intelligence variable comprises two sub-variables: social media exposure and product recommendation—each impacts purchase intention and brand preference, impacting e-customer loyalty. These relationships are summarized in Figure 1.

### 3.4 Cognitive and emotional responses to AI in e-commerce

TAM, together with TRA, deliver useful conceptual patterns regarding consumer actions, yet they fail to represent how AI creates psychological and perceptual effects on user response patterns. A cognitive-affective framework offers a better approach to studying e-commerce customers’ behavior while using AI technology. Consumers understand stimuli through relevance analysis along with an assessment of what is new and what is valuable (Rakib et al., 2022). Cognitive evaluations performed by consumers generate changes to their subsequent attitudes and behaviors. Emotions, including excitement, trust, and satisfaction, together with anxiety, emerge in consumers when AI systems deliver their communications with specific timing and contextual framing (Chekembayeva et al., 2023). AI systems generate product recommendations and social media content that produces positive emotions when customers experience them as relevant, timely, and suited to their context (Bilal et al., 2024). The emotional states generated by AI stimuli act as middle elements between AI stimuli and three behavioral outcomes, including purchase intention, brand attachment and platform loyalty. A dual theoretical perspective of cognition and emotion enhances study foundation because it acknowledges both logical and affective components of AI technology interaction with consumers.

### 3.5 Hypotheses development

#### 3.5.1 Social media exposure positively affects purchase intention

People primarily use social media networks to access e-commerce and discover AI-curated content as their introduction to online stores. The algorithmic feeds on these platforms present consumers with context-specific new products, services, and brands, which enhances brand detection and client interest rates. External stimuli that affect perceived usefulness according to the Technology Acceptance Model eventually influence awareness (Saleem et al., 2022). AI-driven discovery mechanisms on Instagram and TikTok function to boost visibility, which leads to fast product awareness (Onofrei et al., 2022). The continuous nature of mobile-first utilization and real-time marketing techniques found in mobile platforms results in users obtaining more intense exposure to advertisements (Daou and Stephan El Khoury, 2025). Because AI can predict when customers will be most ready to receive content, it can deliver targeted messages that build brand recognition through all customer interactions. For this reason, the study’s hypothesis in this context reads as follows:

*H1*: Social media exposure positively and significantly influences purchase intention.

#### 3.5.2 Product recommendation positively affects purchase intention

Social media content enhanced by AI achieves two outcomes that affect consumer purchase intention by working through the cognitive processes and affective responses. Drawing from the Theory of Reasoned Action, attitudes and perceived norms formed through sustained exposure to persuasive stimuli drive behavioral intentions (Saleem et al., 2022). AI creates content with emotional triggers as well as user-preference adjustments to boost marketing message effectiveness (Bilal et al., 2024). People develop trust and experience authentic perceptions with endorsements from influencers and product evaluations by consumers who share content on platforms that foster buying decisions (Al-Mushayt et al., 2022; Foroughi et al., 2024). Online marketing transitions from passive activity to active consumer intent through AI-personalized content presentation, which builds immediacy and relevance in the current settings. The combination of small platform activities directs customers toward enhanced purchase intent, especially when used in mobile e-commerce environments that move quickly. Hence, our hypothesis in this context reads as follows:

*H2*: Product recommendation positively and significantly influences purchase intention.

#### 3.5.3 Social media exposure positively affects brand preference

Online consumers choose platforms for social interaction and also because these platforms provide relevant commercial content curated by AI technology (Gautam and Sah, 2023). Digital platform selection follows goal-driven behavior that enables users to find environments that address functional and hedonic needs according to the Uses and Gratifications Theory (Marjerison et al., 2022). Users tend to link sophisticated Artificial Intelligence systems in platforms to better align with personal consumption preferences and individual values (Maskuroh et al., 2022; Ratner et al., 2025). Behavioral preferences are strengthened through social media design that merges commercial functionalities using AI technology, which enhances searchability and usability. Platform effectiveness strengthens user loyalty because consumers connect relevance with ease of product engagement (Jenneboer et al., 2022). The repetitious experience of using social media positively influences a user to choose their current platform for maintained engagement. It is for this reason that our hypothesis reads:

*H3*: Social media exposure positively and significantly influences brand preferences.

#### 3.5.4 Product recommendation positively affects brand preference

AI-linked product recommendations transformed from basic static displays into personalized prompt-based suggestions based on user behavior patterns. The systems function to improve both decision-making efficiency while shaping customer brand preference and purchase emotions and brand connections. Advancements in brand perception stem from AI recommendations that base their usefulness and personalization on the Technology Acceptance Model (Nguyen et al., 2022). The AI-driven recommendation system enhances relevance and trust in addition to consumer brand engagement, which results in their acceptance of established brand values (Jin and Zhang, 2025). Consumer appreciation of recommended brands increases through consistent provision of suitable recommendations (Bhattacharya, 2023). Mobile environments experience an increased effect of recommendations because they create contextual, immediate, and immersive suggestions. The meticulously tailored shopping journey created by AI establishes brand preference that goes beyond product benefits because it selects brands through comprehensive customer journeys uplifted by AI enhancement. In summary, we are led to preliminarily believe in the integrity of this concise hypothesis:

*H4*: Product recommendation positively and significantly influences brand preferences.

#### 3.5.5 Purchase intention positively affects e-customer loyalty

The psychological state of consumers willing to buy something represents a fundamental indicator of future customer loyalty and has always proven to be an effective customer behavior predictor. According to the Theory of Reasoned Action, behavioral intentions provide the closest link between actual behavior and brand commitment, along with repeat patronage (Saleem et al., 2022). E-commerce customers who exhibit several consecutive high purchase intentions demonstrate contentment with the shopping platform, which usually develops into heartfelt loyalty and active endorsement (Cardoso et al., 2022). These intentions become stronger because of AI-assisted personalized offers and navigation simplicity together with trust-oriented interfaces, as described in Tena-Monferrer et al. (2022). Consumer affinity to purchase leads to less brand switching since people get comfortable with using the platform’s complete features (Lau et al., 2023). The regular behavioral pattern functions as a fundamental basis for building loyal digital commerce customers. Hence, we make this preliminary claim linking the two variables by stating:

*H5*: Purchase intention positively and significantly influences e-customer loyalty.

#### 3.5.6 Brand preference positively affects e-customer loyalty

The attitudinal attachment that consumers have to a single brand involves quality perceptions alongside identity features and emotional connections. Digital commerce operates with so many choices that brand preference stands as the main loyalty determinant since customers easily move between alternatives. According to consumer behavior theories, specifically the attitude-behavior relationship, consumers who favor a brand tend to show more sustained loyalty behaviors, including continuous purchases and advocacy for the brand (Machi et al., 2022). Consumer satisfaction and loyalty evolve through preference elements, which build trust for customers and decrease competitor attractiveness (Kim et al., 2021; Chiu and Cho, 2021). AI-controlled brand communications, starting from uniform chatbot smartness to intensely tailored content, deeply embed customers into specific brands within their purchasing selection process (Ho and Chow, 2023). The development of loyal brand engagement happens naturally when brand preference grows stronger in terms of emotional connections as well as functional outcomes. Therefore, we find it reasonable to make the preliminary claim:

*H6*: Brand preference positively and significantly influences e-customer loyalty.

## 4 Research methodology

### 4.1 Items measurement and questionnaire design

We adopted a quantitative approach because our data was overly numeric. A quantitative design allowed us to be objective in our analysis and discussion (Gaus, 2017). The study employed a questionnaire as the sole method of data collection. It contained 20 questions following the Likert scale of agreement/disagreement, where 1 represented strong disagreement and 5 represented strong agreement. We obtained the data through an online survey where the questionnaire was hosted on Google Forms. Each variable had a minimum set of three and a maximum of five questions. To ensure the targeted geographical areas were reached, we went to social media sites and targeted specific groups with allegiances with the three cities. Survey questions utilized in the study are presented in Appendix A.

### 4.2 Sampling and data collection

The study considered the Saudi population actively involved in e-commerce and or on social media sites. JCCI (2023) estimates this to be 29.9 million, about 80% of the total Saudi population of 37.47 million. We sampled 157 respondents to represent the population. It included males and females and persons from across the age spectrum to make it more inclusive. Moreover, ethical approval with informed consent was obtained by the deanship of the scientific research committee at Umm Al-Qura University. Additionally, written informed consent for participation in the study has been obtained. The questionnaire has been sent to the respondents through Surveymonkey.com. While it was our interest to sample people from different socio-economic statuses, we found this difficult to achieve, as it was a sensitive piece of information to extract from respondents. Nevertheless, our randomized approach to data collection helped ensure that we obtained respondents from diverse backgrounds. The survey did not differentiate between consumers who used mobile apps and those who used websites because most users moved easily between both platforms. The analysis used a cross-section of typical e-commerce and did not focus on any particular type of product. The researchers adopted this method to preserve general consumer behavior observations, yet this might constrain their ability to discover category-specific insights.

As indicated above, we employed the questionnaire to collect data for this study. In our geographical coverage of the country, we organized it into three regions: East, West, and the Middle. In each region, we selected one city in which we conducted the survey. We selected Al Hofuf from the East, the center - Riyadh, and the West—Madinah. The biggest share of respondents came from Riyadh (55%), followed by Madinah (27%) and Al Hofuf (18%). We deliberately excluded socioeconomic variables, including financial standing and work types, from the dataset because they recognized the ethical concerns about Saudi society’s personal information and the possibility of misrepresenting responses. The pre-study evaluation showed that financial questions caused participants to provide ambiguous data, which threatened the validity of the research data. The questions were purged from the survey because we needed to guarantee better response quality and participant comfort.

### 4.3 Structural model and structural equation modeling (SEM) approach

**Figure 2:**
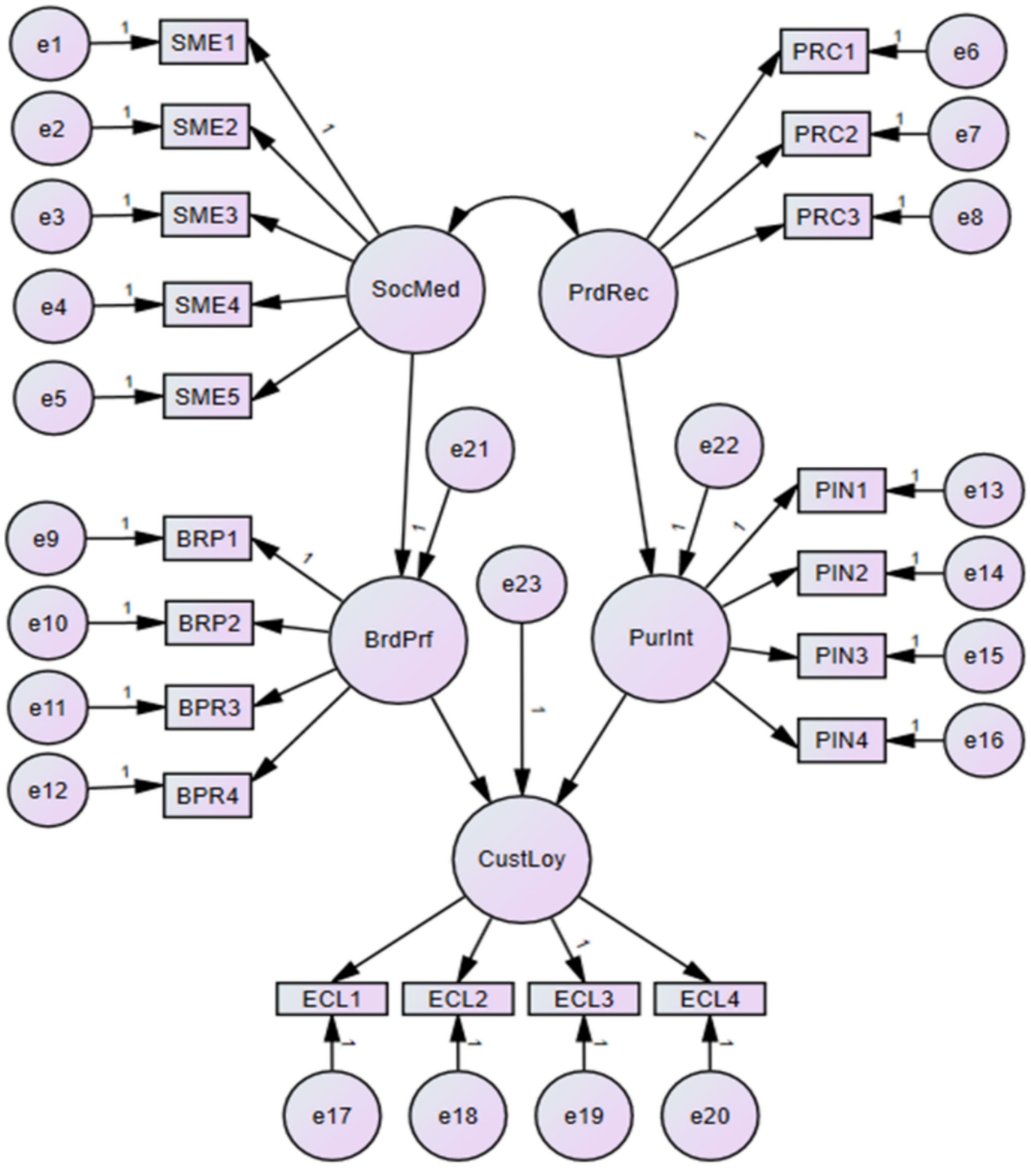
Measurement model.

The structural model originates from two key variables: social media exposure and product recommendation. These two constructs are hypothesized to impact two secondary variables: brand preference and purchase intention. Finally, the structural model will test whether brand preference and purchase intention significantly affect e-customer loyalty. Figure 2 shows the flow of effects throughout the measurement model.

Moreover, one of the key considerations was how we would analyze the findings from the collected data. While there were many options, SEM seemed to be the most comprehensive approach, given our study’s nature and goals. Firstly, our research involved several variables with heavy hypothetical interdependence among them. Secondly, the hypotheses contended by the study postulate a situation in which a multivariate regression and path analysis are inevitable. Structural equation modeling is specifically equipped to handle such analysis scenarios because it can track and compute the significance of path-wise relationships, as envisaged in our model (Sarstedt et al., 2022). Based on this equation modeling, we base our findings and analysis on the next section.

## 5 Results

### 5.1 Demographic analysis

The distribution of sample participants according to demographic features makes it possible to determine the validity of research findings. The respondent sample revealed a dominant number of participants (50.3%) aged 18–25, with 26.1% within the 26–35 age range. A total of 15.3% of participants selected the age range of 36–45 years, and 8.3% belonged to ages over 45. The younger age grouping matches the main customer audience for e-commerce platforms in Saudi Arabia and other markets, given the widespread use of AI shopping technology among digital generations. The survey population showed that the male and female distribution was similar (47.1% females and 44.6% males), and 8.3% refused to reveal their gender. A reasonable amount of sample inclusivity exists, but additional research could enhance gender equality within the sample makeup. More than half of the study participants (54.1%) graduated from college, while another 33.1% earned their college diplomas. Among the respondents, only 10.1% had postgraduate education, and 2.5% had finished high school. The sample data indicates that most participants hold degrees while maintaining a young demographic consistent with the typical audience expected to adopt AI-enhanced e-commerce services (Table 1).

**Table 1:** Demographic information.

Category	Frequency	Percent
Age		
18–25 years	79	50.3
26–35 years	41	26.1
36–45 years	24	15.3
>45 years	13	8.3
Total	157	100.0
Gender		
Male	70	44.6
Female	74	47.1
Undisclosed	13	8.3
Total	157	100.0
Education		
High school	4	2.5
College diploma	52	33.1
Bachelor’s degree	85	54.1
Master’s degree	12	7.6
Ph.D	4	2.5
Total	157	100.0

### 5.2 Assessment of the measurement model

In assessing the measurement model, we examined four critical aspects: indicator reliability, internal consistency, convergent validity, and discriminant validity.

#### 5.2.1 Indicator reliability

This metric is also referred to as composite reliability. Through composite reliability assessment, researchers determine how much each measure within a latent construct correlates with its underlying dimensions and other items. The standard benchmark for composite reliability stands at 0.7, according to Kline (2023), to demonstrate that measurement items properly capture the construct free from substantial errors. The research criteria demonstrated that each measured construct exceeded 0.7 composite reliability. The measurement of e-customer loyalty exhibited the strongest composite reliability score at 0.96 because it demonstrated strong internal relationships among the observed variables. The reliability score of product recommendation reached 0.80 above the minimum threshold, which validated its values. Further inspection of the construct becomes necessary because of its moderate score level. The methodology could have failed to grasp how customers perceive AI predictive recommendations because participants might interpret the recommendations differently, resulting from various factors, including their personal exposure level and trust in algorithmic processes and the quality of personalized recommendations. Future investigation should improve the measurement indicators for construct reliability or expand the scale to represent diverse customer interactions with product recommendation systems.

#### 5.2.2 Internal consistency

Internal consistency measures the stability and coherence of questionnaire items designed to assess a particular construct. Reliability indicates that participants answer related questions in parallel across multiple surveys to strengthen data collection devices. According to Cronbach’s alpha standard, the accepted threshold value for reliability measurement lies at 0.7 or above (Kline, 2023). The findings demonstrate appropriate uniformity among the five construct measurements. Data reliability for E-customer loyalty reached an exceptionally high level (*α* = 0.958), confirming the validity of conclusions concerning consumer loyalty within AI-based e-commerce systems. Cronbach’s alpha for product recommendation amounted to 0.727 while staying within the acceptable range. The acceptable internal consistency measurement reinforces previous results while confirming the construct’s validity even though more enhancement of the construct can potentially yield better outcomes. The established constructs in the literature display higher internal consistency than emerging constructs, such as AI-based recommendation products, because they are better defined and measured in user perception and measurement frameworks.

#### 5.2.3 Convergent validity

The analysis of convergent validity checks how various measurement items that assess a single construct agree in terms of shared variance. The dominant evaluation method for construct indicator convergence is Average Variance Extracted (AVE), which demonstrates sufficient alignment when exceeding 0.5 (Hair et al., 2021). Overall, the convergent validity of the model is confirmed through its average AVE of 0.768 in the current research. The AVE value for product recommendation stands at the lowest point of 0.58 compared to other constructs. This value meets the necessary threshold standard but stands close to the cutoff point, implying limited precision among the construct indicators compared to other variables. The wide-ranging application methods of AI recommendations and user knowledge regarding platform tools are possible explanations for the low AVE scores. Future work must review and improve the measurement scale to understand AI-based recommendations because current indicators might need modifications through contextual user-based elements or machine learning profiling techniques. Future research focus on AI’s effect on e-customer loyalty requires improved construct precision, which could be achieved by resolving current measurement problems (Table 2).

**Table 2:** Construct indicators.

Construct	Items	Factor loading	Composite reliability	Indicators	Cronbach’s alpha	AVE
Social media exposure	SME1	0.916	0.94	5	0.953	0.77
	SME2	0.850				
	SME3	0.767				
	SME4	0.981				
	SME5	0.860				
Product recommendation	PR1	0.766	0.80	3	0.727	0.58
	PR2	0.911				
	PR3	0.559				
Brand preference	BP1	0.824	0.94	4	0.940	0.80
	BP2	0.900				
	BP3	0.871				
	BP4	0.967				
Purchase intention	PI1	0.926	0.95	4	0.932	0.83
	PI2	0.823				
	PI3	0.986				
	PI4	0.893				
E-customer loyalty	ECL1	0.898	0.96	4	0.958	0.86
	ECL2	0.891				
	ECL3	0.912				
	ECL4	0.999				

#### 5.2.4 Discriminant validity

The final aspect of the measurement model’s assessment is discriminant validity. It refers to the extent to which responses to questionnaire construct items are more related to each other than how they relate to items from other constructs (Kline, 2023). The general guideline is that the correlation coefficient between the items of a variable in a questionnaire should be greater than their correlation with other variables. According to this criterion, the instrument was deemed legitimate. Table 3 shows that items from the same constructs appear distinct from those of other constructs by exhibiting a significantly higher correlation.

**Table 3:** Results on discriminant validity.

	SME	PRC	PIN	BPR	ECL
SME	**0.911**				
PRC	0.560	**0.837**			
PIN	0.713	0.846	**0.777**		
BPR	0.623	0.835	0.762	**0.815**	
ECL	0.812	0.807	0.626	0.635	**0.908**

Bold values are higher correlation.

### 5.3 Assessment of the structural model

**Figure 3:**
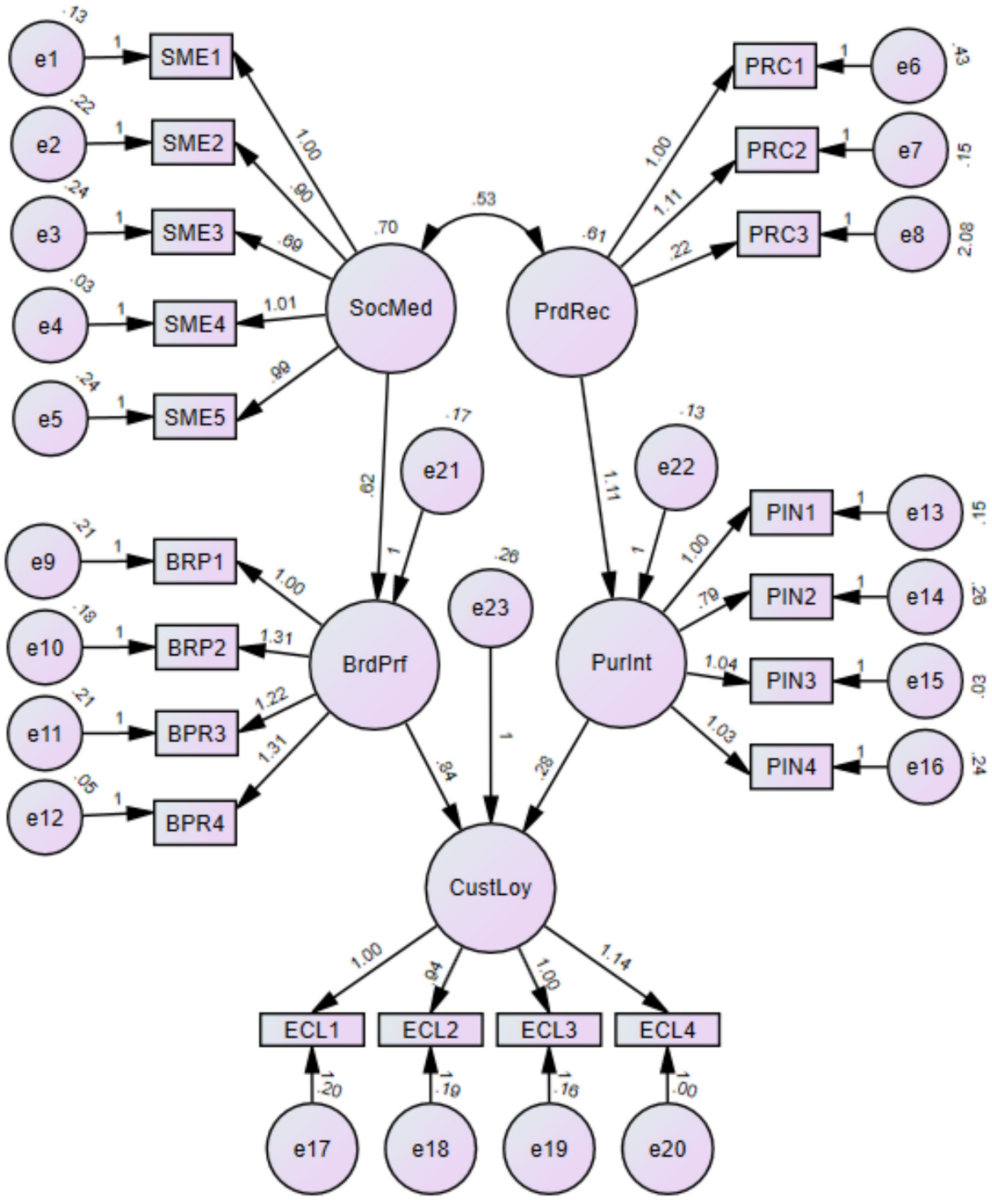
Structural model.

This study’s structural model shows the relationships between and among the construct variables. Figure 3 shows that social media exposure, product recommendation, brand preference, purchase intention, and e-customer loyalty positively affected their respective dependent variables throughout the path diagram.

### 5.4 Model fit indices

The structural model scored a chi-square value of 514.355 on the default model, while the independence model scored 2,848.480, as shown in Table 4. According to Hair et al. (2021), determining whether a model is significant is the default model’s CMIN/DF value. The model’s significance is affirmed if the CMIN/DF value is <5. Our study scored a CMIN/DF value of 3.117, implying that the structural equation model is statistically significant.

**Table 4:** Showing model fit indices.

Model	NPAR	CMIN	DF	*P*	CMIN/DF
Default model	45	514.355	165	0.000	3.117
Saturated model	210	0.000	0.000		
Independence model	20	2848.480	190	0.000	14.992

### 5.5 Hypotheses testing

Ultimately, we conducted several regression tests to ascertain the validity of our hypotheses. In total, we did six tests for the corresponding six hypotheses. Table 5 below summarizes the findings from these tests. Both social media exposure and product recommendation were significantly influential in determining consumer purchase intention and brand preference. They all scored R-squared coefficients >0.4. The latter seemed more effective in influencing the two dependent variables between social media exposure and product recommendation. We examined the effect of purchase intention and brand preference on e-customer loyalty. While both variables has a significant impact, brand preference was more influential. The model was statistically significant from the beta score of 0.505 (*p* = 0.000) and beta score of 0.631 (*p* = 0.000) for the relationship between social media exposure and purchase intention and the relationship between product recommendation and purchase intention, respectively. This means all AI construct variables significantly influenced the purchase intention construct. In addition, the results were statistically significant from the beta score of 0.527 (*p* = 0.000) and beta score of 0.733 (*p* = 0.000) for the relationship between social media exposure and brand preference and the relationship between product recommendation and brand preference, respectively. The last two relationships have also scored significant results with beta 0.686 (*p* = 0.000) and beta 0.686 (*p* = 0.000) for the relationship between purchase intention and e-customer loyalty and the relationship between brand preference and e-customer loyalty, respectively. This means that purchase intention and brand preference significantly influenced the e-customer loyalty construct.

**Table 5:** Hypotheses results.

Hypothesis	R	R-Squared	Adj. R-Squared	*p*-value	Beta	Constant	Decision
H1. SME → PIN	0.670	0.449	0.448	0.000	0.505	1.457	Accept
H2. PRC → PIN	0.718	0.516	0.515	0.000	0.631	1.290	Accept
H3. SME → BPR	0.642	0.412	0.411	0.000	0.527	1.378	Accept
H4. PRC → BPR	0.766	0.587	0.586	0.000	0.733	0.917	Accept
H5. PIN → ECL	0.746	0.556	0.556	0.000	0.686	1.334	Accept
H6. BPR → ECL	0.813	0.661	0.661	0.000	0.686	1.329	Accept

## 6 Discussions, implications, limitations, and conclusion

### 6.1 Discussions and implications

#### 6.1.1 Discussion

The study investigated the effect of artificial intelligence on e-customer loyalty in Saudi Arabia. A purposeful decision was made to include five constructs in the modeling framework: social media exposure, product recommendation, purchase intention, brand preference, and e-customer loyalty based on foundational theories from the Technology Acceptance Model (TAM) and Theory of Reasoned Action (TRA) (Saleem et al., 2022). The model omitted relevant constructs, including trust and satisfaction and perceived service quality, because the researchers aimed to achieve both parsimony and avoid over-specification. The research study maintains solid validity with its Saudi Arabian population yet features specific restrictions stemming from its sample construction. The study shows an excessive number of participants aged 18–25. At the same time, the gender split was nearly even despite its narrow scope regarding socio-economic elements and geographical areas limited to three major urban regions, thus reducing the applicability of its findings. The assessment of AI adoption needs to focus on distinct demographic categories because variables such as digital knowledge, financial capacity, and social customs will define how users accept AI solutions.

The research team described the demographic variables, yet omitted their inclusion in the structural model framework. The researcher chose this approach according to the study’s main goal of examining behavioral and attitudinal constructs corresponding to the Technology Acceptance Model and Theory of Reasoned Action (Saleem et al., 2022). Including demographic variables would have added complexity and unnecessary elements to investigating AI tool-loyalty functional relations. Future researchers should investigate how demographic and socio-economic variables modify the relationships by performing multi-group analysis and stratified modeling.

Findings on the effect of social media (a construct of artificial intelligence) on purchase intention were positive and statistically significant. This notion is consistent with the study by Foroughi et al. (2024), who contends that when customers are exposed to social media, they tend to improve their intention to purchase products via e-commerce. Similarly, our investigation found that social media also positively impacts brand preference. Again, this notion validates the position held by Maskuroh et al. (2022), as the source asserts that content targeting is social media’s most formidable arsenal in influencing consumer tastes and preferences.

The strong connection between social media contact with customers and buying commitment demonstrates that active content delivery strategies directly stimulate consumer purchasing behavior. The cognitive and emotional aspects seem to determine this observed effect (Rakib et al., 2022). Artificial intelligence feed recommendations help customers perceive their match to products better and feel more comfortable with their decisions while streamlining the buying process. Invisible repetition of socially endorsed products from influencers generates emotional responses which include excitement as well as social belonging and trust (Onofrei et al., 2022). Consumers merge these emotional and cognitive responses when they become receptive to purchase requests from companies and generate well-defined buying intentions.

Product recommendation also formed a significant part of the investigation, as it was responsible for two hypotheses. The first hypothesis relating to product recommendation regarded its effect on purchase intention. Here, our findings suggested that more product recommendation activities bring about higher levels of purchase intention from customers. This view is also shared by Jin and Zhang (2025), indicating that product recommendation engines are responsible for many impulse buying instances among online shoppers. One of the hypotheses also captured the effect of product recommendations on brand preference. Our findings suggest that the two share a positive causal relationship where more aggressive product recommendations result in brand preference. Our findings align with the article by Kim et al. (2021), which claims that effective recommendation algorithms can improve brand preference for a sizable proportion of users.

AI adoption leads directly to the advancement of e-customer loyalty through its ability to assess convenience and foster trust and emotional connection (Ifekanandu et al., 2023). Time-driven consumer trust develops through customized suggestions, adaptive chatbots, and sustained brand contact, resulting in devoted return consumers. Customers whose interactions with AI systems instigate a perception of transparency develop stronger loyalty even when no human interacts with them, according to (Ratner et al., 2025), so AI becomes a vital instrument for loyalty conversion approaches.

The study shows that recommendations influence purchase decisions and choice of brands better than the combination of social media exposure. The beta coefficients demonstrate that product recommendation leads to higher purchase intention rates (*β* = 0.631) than social media exposure (*β* = 0.505) does. The strength of product recommendation on brand preference reaches 0.733, while social media exposure achieves only 0.527. The analytics demonstrate that AI-powered recommendations produce the most effective transition of consumer interest into actual purchase decisions, although social media creates essential initial visibility and engagement. When customers receive personalized recommendations, their cognitive assessment, along with affective bonding, rises to produce stronger commitment and preference (Habil et al., 2023). The distinction between social media visibility and AI recommendation systems determination holds great importance for practitioners since visibility remains essential, but investing in recommendations produces stronger behavioral impacts throughout the complete customer path.

This study demonstrates that product suggestions and social media awareness strongly drive customers toward both buying decisions and brand preference selections since personalized data strategies drive loyalty development in e-customers. The relationships would achieve greater detail when sentiment analysis and customer segmentation analytics are integrated through Artificial Intelligence. According to Huang et al. (2023), the processing system of sentiment analysis lets businesses interpret emotional feedback immediately to help them create smarter recommendation systems along with better marketing content. According to Bhagat et al. (2023), electronic commerce platforms use customer clustering algorithms to split users into dynamic segments based on their behavior patterns and historical interactions; thus, they can develop loyalty strategies for different audience types. This study demonstrates the critical role of AI-driven engagement. However, it failed to specifically identify underlying mechanisms because advanced techniques have the potential to act as strong mediators for improving predictive accuracy and customer retention durations.

E-customer loyalty was the ultimate dependent variable in our study. Brand preference and purchase intention were the independent variables that hypothetically influenced it in our model. We found credible statistical evidence to support the hypothesis that brand preference affects e-customer loyalty. Our study found that a better level of brand preference from customers builds up situations that often lead to more repeat purchases and referrals. This notion is also shared by Hsu et al. (2021), stating that loyalty programs are more effective when aligned with existing brand preferences. Regarding the effect of purchase intention on e-customer loyalty, our study found a statistically significant positive relationship between the two. This relationship is also depicted in the study by Ahmad and Akbar (2023). Its findings concur with ours, as the source finds that e-commerce platforms exploit customers’ purchase intents to ensure that customers remain faithful to the businesses and their products.

Consumer feedback toward automation increasingly determines how AI adoption level affects customer loyalty. Our study suggests that perceived relevance, convenience, and real-time assistance delivered through AI-powered systems enabled Saudi e-commerce retailers to improve their businesses by employing AI systems. This results in improved customer retention in the Saudi e-commerce market. Trust, brand attachment, and satisfaction are loyalty ingredients that tend to grow with a heightened usage of artificial intelligence systems in retail (Helmi et al., 2024). Youthful consumers and digitally proficient members of society exhibit repeated buying patterns from AI-driven systems that make predictive user choices and enhance decision processes. Saudi consumers share some characteristics with global markets regarding AI adoption but show both enthusiasm for AI technology and prudence in their approach to its use. Research by Jenneboer et al. (2022) and Rana et al. (2022) indicates that Gulf market consumers accept automated systems better than Western users, as they are more trusting relative to their Western counterparts. AI applications become effective in building loyalty programs within this favorable context as long as users feel that their data is maintained securely and transparently. Although the essential principles behind AI-driven loyalty remain consistent across all areas, the specific adoption and effects need to be interpreted based on how consumers trust AI and how well it fits their culture.

#### 6.1.2 Implications

This research demonstrates that artificial intelligence strongly affects Saudi Arabian e-customer loyalty through two key mechanisms: social media exposure and product recommendation services. Targeted social media content shows a positive relationship that drives consumers toward purchasing items and selecting particular brands. Products recommended through AI systems boost purchase appeal and brand affinity, frequently triggering customers to buy impulsively (Bhagat et al., 2023). Customer purchase intentions prove fundamental for e-commerce platforms to build e-customer loyalty because brand preference operates as a direct pathway to loyalty. The research delivers essential insights that e-commerce platforms and marketing practitioners in Saudi Arabia must use because they aim to strengthen customer connections in their digital markets.

The results from this study can directly support businesses that wish to use artificial intelligence-controlled loyalty systems that customize real-time interactions with each customer. Organizations should utilize adaptive incentive structures to deliver individually tailored rewards that consider user activities and shopping habits to drive customer return business. Through dynamic pricing algorithms, AI can modify product prices according to demand patterns, user location, and device type, increasing perceived value and urgency (Kim et al., 2021). The analytical capabilities of sentiment analysis and predictive modeling allow Noon and Amazon.sa to develop customized marketing material that enables automated retargeted advertising and personalized digital communications (Al-Mushayt et al., 2022). A combination of AI tools applied within loyalty platforms enables businesses to create loyal customer relationships that substitute short-term transactions. The strategic importance of AI keeps growing since it serves both as a technological capability and commercial enabler in emerging e-commerce systems.

Market-specific recommendations are only one component of AI-enabled business operations, as this technology enables organizations to automatically deploy loyalty scoring mechanisms that monitor customer value and involvement dynamics. Behavioral analytic models for predicting customer loss would allow businesses to discover potential cases of defection at an early stage, generating intervention strategies to retain clients (Cheng et al., 2023). AI tools automatically optimize promotional strategies through online campaign monitoring, which enables businesses to create targeted offers for customer groups according to predicted response patterns (Ho and Chow, 2023). The development of these applications shows how artificial intelligence transitions from being a recommending technology to becoming a fundamental management tool for complete customer relationship management.

The implementation of AI-driven social media recommendations and product suggestions leads to improved purchase behavior along with new operational and ethical obstacles. The primary worry today revolves around privacy matters since users need full disclosure about how their online activities and network behavior are gathered and applied to marketing purposes (Bhattacharya, 2023). Systems using AI try to reinforce confirmation bias while simultaneously restricting access to content variety which results in actual discovery and selection reduction (Bhagat et al., 2023). Attempts to continuously expose users to promotional content reduce their trust in platform authenticity and, in turn, diminish trust in brand authenticity. Marketers need to maintain an equilibrium between campaign optimization and consumer intrusiveness through data transparency services, specific opt-in alternatives, and built-in ethical AI protection during campaign implementation.

### 6.2 Contributions of the study

The research presents significant additions to scholarly knowledge and practical e-commerce strategies regarding AI-based consumer interaction within Saudi Arabian market conditions. The research demonstrates an empirically verified structural model that links social media exposure and product recommendation features of AI to purchase intention, brand preference, and e-customer loyalty. The multi-path structure promotes current discussions by illustrating how AI affects both transactional conduct and the formation of strong brand bonds. The study recognizes the impact of AI-powered systems on local Middle Eastern consumer choice processes, on which research literature seldom focuses. Although AI was not a statistical tool for data collection or validation methods, the findings demonstrate that future research should use AI-improved adaptive surveys and machine-learning sentiment analytics to boost model reliability and accuracy (Hair et al., 2021). The study presents dual methodological significance through its framework integration of the Technology Acceptance Model and the Theory of Reasoned Action for studying consumer behavior with AI-powered platforms that generate loyalty effects.

This study promotes the development of creative artificial intelligence applications for measurement and analytical work. Machine learning applications that use automated bias detection, adaptive questionnaire logic, and real-time anomaly recognition would improve data reliability and integrity in future explorations. Implementing these tools enables automatic adjustments based on respondent behaviors while identifying inconsistencies, which supports highly valid research design approaches in consumer behavior and loyalty modeling. Future measurement processes can include AI-enhanced tools, as it would establish their implementation path. Such machine learning techniques adapt survey questions dynamically to spot inconsistent data with automatic anomaly detection capabilities. The findings in this study can be used to enhance consumer research through increased model precision and validity.

### 6.3 Limitations

One of the limitations of this study is the reliance on self-reported data. Our approach entailed issuing respondents with questionnaires and having them complete their responses by carrying out a self-assessment. Reliance on self-reported measures (like surveys) can introduce bias, as respondents might not always provide accurate or honest answers.

Another limitation is that the investigation anchors itself on the currently available and predominant technological sophistication. Such infrastructure is highly dynamic and may cause the findings to be different if there are major shifts in AI technologies’ future adoption, use, and acceptability. Reliance on self-reported measures (like surveys) can introduce bias, as respondents might not always provide accurate or honest answers.

Another weakness of this study emerges from the inadequate examination of AI-based personalization approaches, including collaborative filtering and natural language processing. The model does not differentiate between these tools, which heavily impact user interaction and loyalty, though they were not separated as distinct components. Future studies should study these research areas through more comprehensive approaches using mixed methodologies that analyze behavioral data while obtaining user emotional feedback.

Additionally, future studies should research how people utilize AI differently on mobile and web platforms because mobile shopping users tend to connect more with social media AI tools, and website users lean toward using AI filtering and search prediction capabilities. This study contributes to Gulf Region AI consumer engagement literature while setting a conceptual base for worldwide e-commerce trade analysis of artificial intelligence loyalty development patterns.

The research also has a limitation because it lacks controls for product type variations between fashion items, electronics and home goods. Consumer responses to AI systems and their buying practices depend on the type of product they purchase especially in terms of purchase speed and building trust and adjusting prices. The research seeks broad e-commerce patterns, but future studies should study specific market segments to increase context clarity. The analysis of identical artificial intelligence mechanisms interacting with different product categories would generate a better understanding of consumer behavior during interactions.

### 6.4 Conclusion

The study conclusively demonstrates that artificial intelligence, notably through social media exposure and product recommendations, significantly influences e-customer loyalty in the Saudi Arabian market. It highlights the significant impact that artificial intelligence has on customer behavior, specifically in terms of increasing purchase intent, brand preference, and, ultimately, customer loyalty. These observations are valuable for organizations seeking to enhance digital visibility and customer interaction methods. Further research is warranted to investigate the enduring consequences of artificial intelligence (AI) on consumer behavior. It scrutinized the ramifications of AI across various demographic groups and assessed the potential hazards and ethical implications linked to AI-powered marketing strategies. Moreover, conducting comparative analyses in various economic and cultural settings would contribute to a more comprehensive comprehension of how AI influences worldwide e-commerce patterns.

## Data Availability

The raw data supporting the conclusions of this article will be made available by the authors, without undue reservation.
