# Supplementary material for: Assessing artificial intelligence’s impact on e-customer loyalty in the Saudi Arabian market
Source: Front Artif Intell. 2025 Apr 30;8:1541678. doi: 10.3389/frai.2025.1541678 (PMC12075180; doi:10.3389/frai.2025.1541678)
Supplement: Supplementary file 1 [file Table_1.docx]

# **Appendices**

## **Appendix A.**

Table Appendix 1: The Questions Used in the Survey.

| **Construct** | **Questions** | **Source** |
| --- | --- | --- |
| **Social Media Exposure** | I frequently encounter advertisements for products and services on social media. | [58] |
|  | I often learn about new products by seeing them on social media. | [58] |
|  | Social media influences my decisions about trying new products. | [58] |
|  | I trust the product information I receive through social media. | [21] |
|  | My shopping habits are influenced by the brands and products I see on social media. | [26] |
|  | | |
| **Product Recommendation** | The product recommendations I receive online are usually relevant to my interests. | [56] |
|  | I appreciate receiving personalized product recommendations when shopping online. | [2] |
|  | AI-generated product recommendations have introduced me to products that I ended up purchasing. | [5] |
|  | | |
| **Purchase Intention** | I plan to purchase products I have seen advertised on social media. | [42] |
|  | Product recommendations significantly increase my intention to make a purchase. | [15] |
|  | I am likely to purchase a product if it has been positively reviewed online. | [27] |
|  | Exclusive online offers and discounts are strong influences on my intent to purchase. | [24] |
|  | | |
| **Brand Preference** | I have specific brands that I prefer to purchase from when shopping online. | [59] |
|  | A brand's social media presence can influence my preference for their products. | [59] |
|  | The quality of a brand's products is a crucial factor in my preference for them. | [58] |
|  | Customer reviews and ratings are important in shaping my preference for a brand. | [58] |
|  | | |
| **E-Customer Loyalty** | I frequently revisit the same e-commerce platforms to make purchases. | [37] |
|  | I would recommend my preferred e-commerce platforms to others. | [60] |
|  | I am satisfied with the customer service provided by my primary e-commerce platforms. | [60] |
|  | Loyalty rewards and programs significantly influence my decision to keep purchasing from the same platform. | [37] |

## **Appendix B:**

Table Appendix 2: Scale of Mean Interpretation

| **Mean Range** | **Interpretation** |
| --- | --- |
| 1.00 – 1.79 | Strongly Disagree |
| 1.80 – 2.59 | Disagree |
| 2.60 – 3.39 | Neutral / Undecided |
| 3.40 – 4.19 | Agree |
| 4.20 – 5.00 | Strongly Agree |
